# TESTING ERADAR: A NEW EHR-BASED ALGORITHM TO INCREASE DEMENTIA RECOGNITION IN HEALTH CARE SYSTEMS

**DOI:** 10.1093/geroni/igae098.1927

**Published:** 2024-12-31

**Authors:** Sascha Dublin, Leah Karliner, Yates Coley, Deborah King, Clarissa Hsu, Sei Lee, Judith Walsh, Deborah Barnes

**Affiliations:** Kaiser Permanente Washington Health Research Institute, Seattle, Washington, United States; University of California San Francisco, San Francisco, California, United States; Kaiser Permanente Washington Health Research Institute, Seattle, Washington, United States; Kaiser Permanente Washington Health Research Institute, Seattle, Washington, United States; Kaiser Permanente Washington, Seattle, Washington, United States; University of California San Francisco, San Francisco, California, United States; University of California San Francisco, San Francisco, California, United States; University of California San Francisco, San Francisco, California, United States

## Abstract

Electronic health records (EHRs) hold potential for identifying individuals with undiagnosed dementia. Using machine learning, our team developed and externally validated a predictive algorithm called the EHR Risk of Alzheimers and Dementia Assessment Rule (eRADAR) that estimates the likelihood an individual has undiagnosed dementia with high accuracy (C statistics of 0.79 to 0.84). Through two embedded pragmatic trials, we are implementing eRADAR in 11 primary care clinics within Kaiser Permanente Washington and University of California, San Francisco. The target population is older adults age 65+ without a documented dementia diagnosis or medication. Primary care providers (PCPs) are randomized to intervention or usual care. The eRADAR algorithm is used to identify individuals with eRADAR scores in the top 15-20%, who are invited to a “brain health” visit that includes assessment of instrumental activities of daily living, depressive symptoms, and cognitive function (Montreal Cognitive Assessment). Results are shared with the patient and PCP, and the PCP is responsible for making the final diagnosis (with decision support provided through the EHR). To date, we have implemented the intervention in 9 clinics and conducted over 590 brain health visits. About 31% (658/2137) of high-risk individuals accept a brain health visit. Of these, about 18% have results suggesting dementia and another 30% mild cognitive impairment. Post-visit surveys show high acceptance of and satisfaction with the intervention. The primary study outcome is rate of dementia diagnosis over 12-month follow-up (completed by April 2025). We are also conducting semi-structured interviews to illuminate benefits and harms.

